# Increased Soluble CD155 in the Serum of Cancer Patients

**DOI:** 10.1371/journal.pone.0152982

**Published:** 2016-04-06

**Authors:** Akiko Iguchi-Manaka, Genki Okumura, Hiroshi Kojima, Yukiko Cho, Rei Hirochika, Hiroko Bando, Toyomi Sato, Hiroyuki Yoshikawa, Hisato Hara, Akira Shibuya, Kazuko Shibuya

**Affiliations:** 1 Department of Breast and Endocrine Surgery, Faculty of Medicine, University of Tsukuba, Tsukuba, Japan; 2 Immunology, Faculty of Medicine, University of Tsukuba, Tsukuba, Japan; 3 Ibaraki Clinical Education and Training Center, University of Tsukuba Hospital, Tsukuba, Japan; 4 Department of Clinical Oncology, Ibaraki Prefectural Central Hospital, Kasama, Japan; 5 Obstetrics and Gynecology, Faculty of Medicine, University of Tsukuba, Tsukuba, Japan; 6 Japan Science and Technology Agency, Core Research for Evolutional Science and Technology (CREST), University of Tsukuba, Tsukuba, Japan; 7 Life Science Center of Tsukuba Advanced Research Alliance (TARA), University of Tsukuba, Tsukuba, Japan; 8 AMED-CREST, AMED, Japan Agency for Medical Research and Development, Tokyo, Japan; Mie University Graduate School of Medicine, JAPAN

## Abstract

Emerging evidence suggests that DNAM-1 (CD226) play an important role in the recognition of tumor cells and their lysis by cytotoxic T lymphocytes (CTL) and NK cells. Although the DNAM-1 ligand CD155 is ubiquitously expressed in various tissues, many human tumors significantly upregulate the expression of CD155; DNAM-1 on CTL and NK cells may be involved in tumor immunity. However, unlike those in mice, human tissues also express soluble isoforms of CD155 (sCD155) that lack the transmembrane region. Here, we show that sCD155 levels were significantly higher in the sera of 262 patients with lung, gastrointestinal, breast, and gynecologic cancers than in sera from healthy donors. In addition, the sCD155 levels were significantly higher in patients with early stage (stages 1 and 2) gastric cancer than in healthy donors, and were significantly higher in patients with advanced stage (stages 3 and 4) disease than in patients in those with early stage disease and healthy donors. Moreover, the sCD155 levels were significantly decreased after surgical resection of cancers. Thus, sCD155 level in serum may be potentially useful as a biomarker for cancer development and progression.

## Introduction

Immune surveillance of tumors suppresses cancer development to protect the host. Key players in cell-mediated immunity to tumors, cytotoxic T lymphocytes (CTL) and NK cells [1, 2], mediate tumor recognition and activation through their antigen receptors and a variety of adhesion and costimulatory molecules [2, 3]. Interactions of cell surface receptors with their ligands expressed on tumor cells induce cytotoxic activity of CTL and NK cells against tumors [4].

DNAM-1 (CD226) is a member of the immunoglobulin superfamily and is expressed on NK cells, T cells, monocytes, macrophages, and platelets [5, 6]. Its ligands in humans and mice are the poliovirus receptor CD155 and its family member CD112 (PPR-2 [PVR-related family 2], also called nectin-2) [7–9]. Human CD155 and CD112 are broadly distributed on epithelial and endothelial cells in many tissues [10, 11]; notably, they are overexpressed on various tumors, including colorectal [12, 13], gastric [12], and ovarian cancers [14]; neuroblastoma [15]; myeloid leukemias [16]; multiple myeloma [17]; and melanoma [18]. Interactions between CD155 and CD112 on tumor cells and DNAM-1 on NK and T cells augment cell-mediated cytotoxicity and cytokine production [7, 8]; DNAM-1 is likely involved in immunity against CD155- and CD112-expressing malignant tumors. In fact, in a model of chemically induced tumors in DNAM-1-deficient mice, DNAM-1 is important for immune surveillance against CD155-expressing tumors [19]. Therefore, CD155 on tumors is crucial for DNAM-1-mediated tumor immunity.

However, in addition to membrane-bound CD155 (mCD155, CD155α), human tissues (unlike those in mice) express soluble CD155 (sCD155) (CD155β and CD155γ) encoded by splicing isoforms of *CD155* that lack the transmembrane region [20, 21]. Here, we examined the serum levels of sCD155 in 262 patients with variable cancers and show that sCD155 may be useful as a biomarker for cancer development and progression.

## Materials and Methods

### Cell lines

We used the following human cell lines obtained from ATCC: HeLa (cervical carcinoma), HOS (osteosarcoma), RD (rhabdomyosarcoma), U87MG (glioma), Jurkat (T cell leukemia), and Colo 205 (colorectal carcinoma). MethA (methylcholanthrene-induced sarcoma from BALB/c mouse) was provided by Dr. Eiichi Nakayama (Okayama University).

### Samples

Tissue samples were obtained from primary cancer patients who underwent surgical resection at the University of Tsukuba Hospital, Japan. Serum samples were obtained from primary cancer patients at University of Tsukuba Hospital and Ibaraki Prefectural Central Hospital, Japan, and from healthy volunteers. Written informed consent was obtained from all patients and healthy volunteers. This study was approved by the ethics committee of the University of Tsukuba and the Ibaraki Prefectural Central Hospital (Approval number 531–5 and 307). Disease stage was classified according to The Union for International Cancer Control (UICC) TNM Classification of Malignant Tumours.

### Mice

BALB/c mice were purchased from Charles River (Yokohama, Japan). All mice were housed and bred under specific pathogen-free conditions at the Animal Resource Center of the University of Tsukuba. Experimental mice were used at 7–10 weeks of age. All experiments using mice were approved by the Animal Experiment Committee of the University of Tsukuba (Approval number 09–390 and 10–237) and performed according to the guidelines of the Animal Experiment Committee of the University of Tsukuba.

### PCR

Total RNA was extracted from cell lines and tissues with Isogen reagent (Nippon Gene). For RT-PCR, we used High-Capacity cDNA Reverse Transcription Kits (Applied Biosystems). PCR analysis of *CD155* splicing variants was performed, as previously described [21].

### Quantitative real-time PCR

Total RNA was extracted from tissues by using Isogen reagent (Nippon Gene). qRT-PCR analysis of *CD155* splicing variants was performed with TaqMan Gene Expression Assays (Applied Biosystems) and Applied Biosystems 7500 Fast Real-Time PCR System. We used the following TaqMan Gene Expression Assays: Hs1050633_m1 (*CD155α*), Hs1050636_m1 (*CD155γ*), and Hs99999903_m1 (*ACTB*). For *CD155β*, we used custom TaqMan Gene Expression Assays with the following primers and reporter: forward primer 5’-AAAGAGGGACCTCCCAGTGA-3’, reverse primer 5’-GAATAGGAGACATGCCCATTAGCT-3’, and reporter 5’-CACTCAGGTACAGAGCATG-3’. By using an Agilent 2100 Bioanalyzer, we analyzed the quality of total RNA for qRT-PCR for an RNA integrity number > 7. All values were determined in triplicate.

### ELISA for human soluble CD155

sCD155 levels in sera were measured by sandwich ELISA using mouse anti-human CD155 mAb (TX24) and rabbit anti-human CD155 polyclonal Ab (pAb) as capture and detection Abs, respectively, followed by HRP-linked anti-rabbit IgG (GE Healthcare) and QuantaBlu Fluorogenic Peroxidase substrate (Pierce Biotechnology). Black 96-well plates (Greiner Bio-One) were coated with mouse anti-human CD155 mAb (TX24, 2 μg/mL blocking buffer, 100 μL/well) for capture overnight at 4°C, blocked with blocking buffer (10% FBS, 200 μL/well) for 1 h at room temperature, and washed three times with washing buffer (0.05% Tween 20). Human CD155-Fc fusion protein (as standards) and serum samples were plated at 100 μL/well, incubated for 1 h at room temperature, and washed with washing buffer. After incubation under the same conditions with 100 μL of rabbit anti-human CD155 pAb (5 μg/mL in blocking buffer), washed plates were incubated under the same conditions with 100 μL of anti-rabbit IgG-HRP (1:2000 in washing buffer), washed, and reacted with 100 μL of QuantaBlu Working Solution (Pierce Biotechnology) for 30 min at room temperature. We stopped the reactions with 100 μL of QuantaBlu Stop Solution (Pierce Biotechnology) and measured the relative fluorescence unit (RFU) of each well at wavelengths of 320 nm for excitation and 420 nm for emission using a Spectra Max M2e reader (Molecular Devices). All values were determined in triplicate. The mouse anti-human CD155 mAb (TX24) and human CD155-Fc fusion protein were generated in our laboratory as previously described [8]. The rabbit anti-human CD155 pAb was generated in our laboratory by standard methods.

### Establishment of MethA transfectant expressing mouse sCD155

The cDNA encoding extracellular region of mouse CD155 was subcloned into the p3×FLAG-CMV-13 expression vector (Sigma-Aldrich) and transduced into MethA cells to generate transfectant stably expressing Flag-tagged sCD155 using DMRIE-C transfection reagent (Invitrogen). The transfectant was selected by G418 (Sigma-Aldrich) and passaged in the peritoneal cavity of mice.

### ELISA for mouse CD155-3×FLAG fusion protein

Flag-tagged sCD155 in mouse ascites and serum were measured by sandwich ELISA using a rat anti-mouse CD155 mAb (TX56) and mouse anti-FLAG BioM2 mAb (Sigma-Aldrich) as capture and detection mAbs, respectively, followed by streptavidin-HRP conjugate (GE Healthcare) and QuantaBlu Fluorogenic Peroxidase substrate (PIERCE). Black 96-well plates (Greiner Bio-One) were coated with rat anti-mouse CD155 mAb (TX56, 2 μg/mL in blocking buffer (10% FBS in PBS), 100 μL/well) overnight at 4°C, treated with the blocking buffer (200 μL/well) for 1 h at room temperature, and washed three times with a washing buffer (0.05% Tween 20). Samples were plated at 100 μL/well, incubated for 1 h at room temperature, and washed with the washing buffer. After incubation under the same conditions with 100 μL of anti-FLAG BioM2 (Sigma-Aldrich, 1 μg/mL blocking buffer), plates were incubated under the same condition with 100 μL of streptavidin-HRP (GE Healthcare, 1:2000 washing buffer), washed, and reacted with 100 μL of QuantaBlu Working Solution (Pierce Biotechnology) for 30 min at room temperature. After stopping the reactions with 100 μL of QuantaBlu Stop Solution (Pierce Biotechnology), we measured relative fluorescence units of each well at wavelengths of 320 nm for excitation and 420 nm for emission using a Spectra Max M2e (Molecular Devices). All values were determined in triplicate. Rat anti-mouse CD155 mAb (TX56) was generated in our laboratory as previously described [8].

### Tumor growth assay

Mice were inoculated subcutaneously in the back with 8 × 10^4^ sCD155-MethA cells. Mice were monitored for tumor size (the long (L) and short (S) dimensions) by using calipers once a week, and the tumor volumes were calculated by the equation: volume = (L × S^2^) / 2, as previously described [22]. Mice were euthanized by CO2 or anesthesia after the end of the experiment.

### Statistics

Statistical analyses were performed by using the two-tailed Mann–Whitney U test and the two-tailed Student t test.

## Results

### Expression of CD155α and CD155β in tumor tissues was higher than that in non-tumor tissues

**Fig 1:**
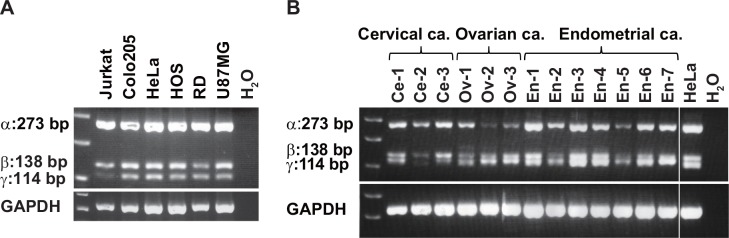
Human cancer cell lines and tissues express sCD155 RNA. PCR was performed using the primer sets located on each side of the different splice sites. Three bands of predicted sizes (α: 273 bp, β: 138 bp, γ: 114 bp) are observed in PCR products. mRNA for membrane-bound (α) and soluble *CD155* (β, *γ*) was expressed by various human cancer cell lines (**A**) and cervical, ovarian, and endometrial cancer tissues (**B**).

**Fig 2:**
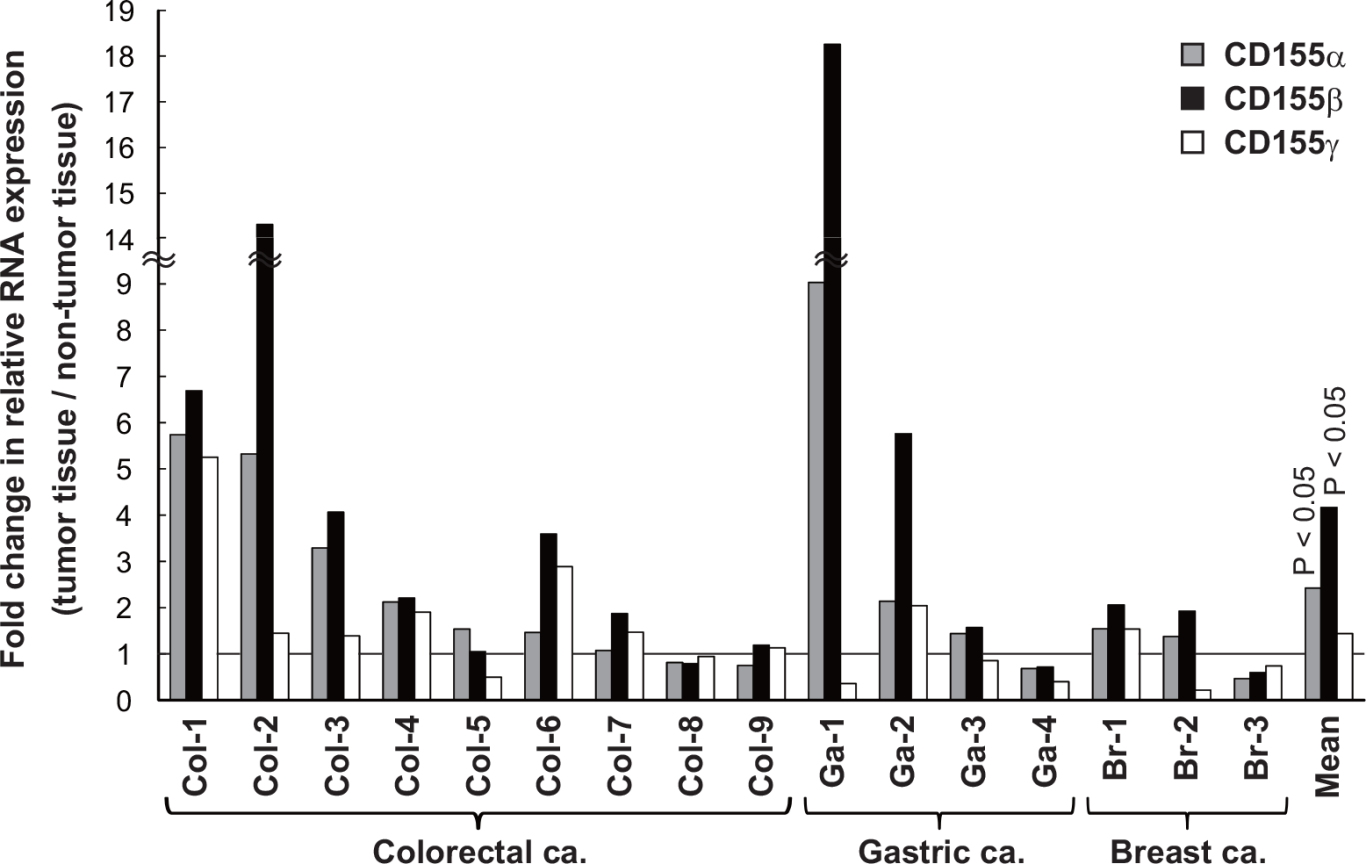
Expression of CD155α and CD155β in tumor tissues was higher than that of non-tumor tissues. Tumor and adjacent non-tumor tissues were taken from surgical resection specimens of colorectal (adenocarcinoma, n = 9), gastric (adenocarcinoma, n = 4), and breast (invasive ductal carcinoma, n = 3) cancers. qRT-PCR of *CD155* RNA splicing variants was performed, and the fold change of relative expression of the tumor tissue versus the adjacent non-tumor tissue was calculated. The expression of membrane-bound (α) and soluble (β) *CD155* RNA in the tumor tissues was significantly higher than that of the non-tumor tissues (P < 0.05).

Although sCD155 is highly expressed in colorectal cancers [12], its expression profile in other cancers is unclear. By RT-PCR, we analyzed the expression of *CD155* mRNA in several tumor cell lines and primary cervical, ovarian, and endometrial cancers; all of the cell lines and cancers expressed both membrane-bound (*CD155α*) and soluble (*CD155β* and *CD155γ*) *CD155* mRNA (Fig 1). Then, by quantitative real-time PCR (qRT-PCR), we compared the expression of *CD155* isoforms among colorectal, gastric, and breast cancers and their adjacent non-tumor tissues; the expressions of *CD155α* and *CD155β*, but not *CD155γ*,were significantly higher in the cancers than in the non-tumor tissues (P < 0.05) (Fig 2).

### sCD155 levels were higher in sera of cancer patients than that in those of healthy donors

**Fig 3:**
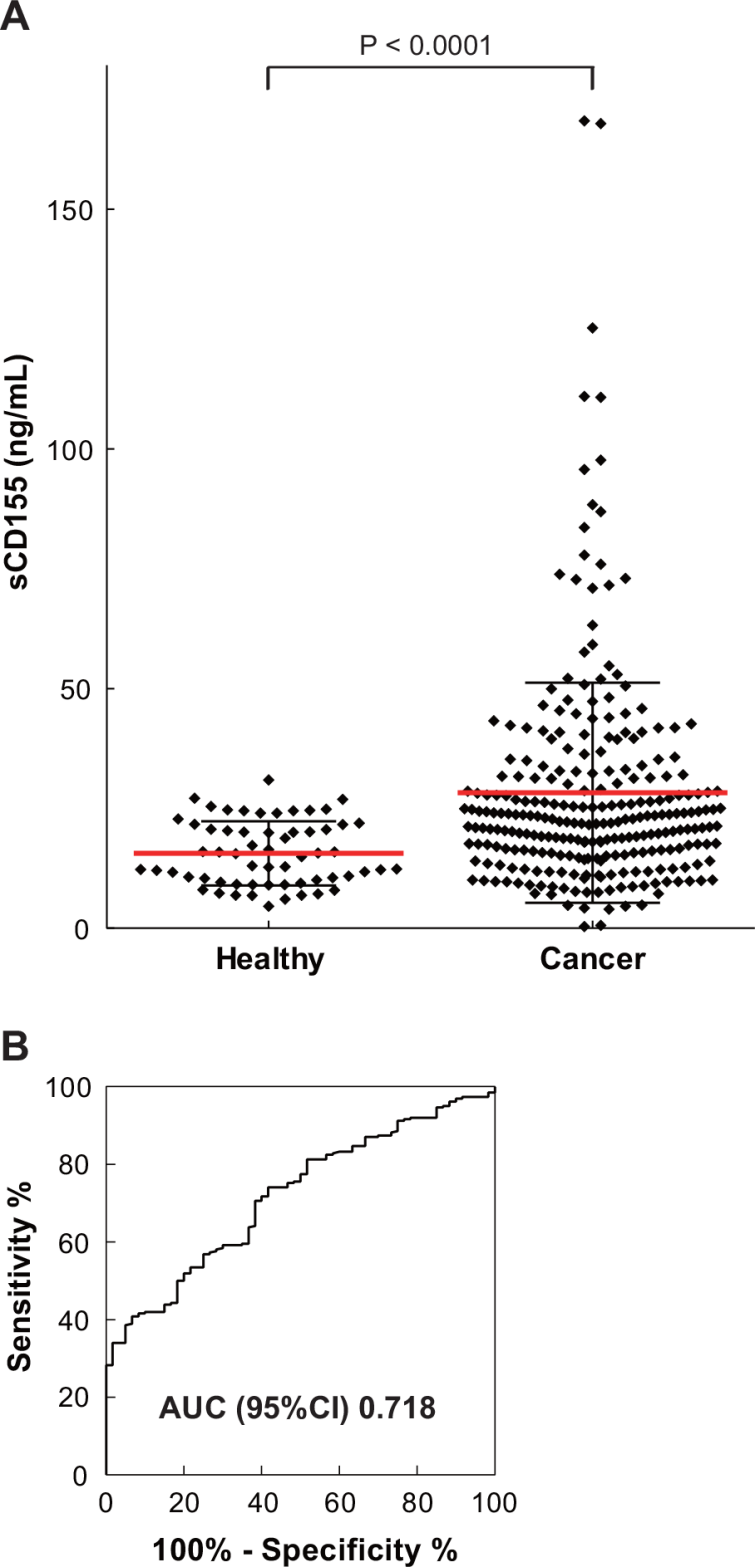
Soluble CD155 levels in sera of cancer patients showed higher than that in sera of healthy donors. (**A**) Soluble CD155 levels in sera of healthy donors (n = 60) and cancer patients (n = 262) were analyzed by sandwich ELISA. Compared with healthy donors, the sera of cancer patients had significantly higher sCD155 levels (P < 0.0001). Red bar: mean, black bar: SD. (**B**) Receiver operating characteristic-curve illustrating the power of soluble CD155 level to discriminate between healthy donors and cancer patients.

Because the expression of *sCD155* in cancer tissues was upregulated, we established a sandwich ELISA for measuring sCD155 (S1 Fig) and quantified sCD155 in the sera of 262 patients with various cancers, including lung, esophageal, gastric, colorectal, bile-duct, pancreatic, breast, ovarian, endometrial, and cervical cancers (Table 1). Compared with healthy donors (n = 60), the sera of cancer patients had significantly higher sCD155 levels (mean = 15.6 ng/mL and 28.3 ng/mL, respectively, P < 0.0001) (Table 1, Fig 3A), which were not affected by patient age or gender (data not shown). The receiver operating characteristic curve illustrates the power of the sCD155 level for discriminating between cancer patients and healthy donors; the value for the area under the curve was 0.718 (Fig 3B).

**Table 1:** Clinical parameters and level of sCD155 in healthy donors and cancer patients.

		Number (male/female)	Mean age	Stage 0/I/II/III/IV/unk	sCD155 level (ng/mL)			P value
					Mean	Median	Range	
Healthy controls		60 (29/31)	57.8	-	15.6	15.3	4.6–30.9	-
All cancer patients		262 (122/140)	63.2		28.3	22.8	0.3–168.5	<0.0001
	Lung cancer	52 (35/17)	68.5	2/36/ 7/ 4/ 3/ 0	23.2	19.1	0.3–97.7	<0.05
	Esophageal cancer	8 (8/0)	69.9	0/ 0/ 3/ 2/ 3/ 0	35.3	26.1	11.8–111.0	<0.001
	Gastric cancer	49 (37/12)	66.5	0/19/ 5/ 5/20/ 0	25.0	20.6	8.7–73.8	<0.0001
	Colorectal cancer	12 (4/8)	63.6	0/ 0/ 1/ 4/ 7/ 0	32.0	25.2	7.5–95.7	<0.001
	Bile-duct cancer	25 (16/9)	72.0	0/ 1/ 1/ 1/17/ 5	39.5	39.9	14.7–77.9	<0.0001
	Pancreatic cancer	18 (14/4)	62.1	0/ 0/ 0/ 1/17/ 0	46.4	27.2	11.1–168.5	<0.0001
	Breast cancer	32 (0/32)	53.6	2/10/12/ 4/ 4/ 0	21.3	20.5	4.2–50.6	<0.05
	Ovarian cancer	23 (0/23)	57.7	0/12/ 1/ 7/ 3/ 0	26.2	23.2	4.8–125.3	<0.01
	Endometrial cancer	16 (0/16)	59.5	0/10/ 1/ 3/ 2/ 0	26.3	24.6	4.6–71.6	<0.01
	Cervical cancer	15 (0/15)	49.6	0/ 4/ 6/ 4/ 1/ 0	19.6	20.4	7.5–39.6	NS
	Others	12 (8/4)	63.8		41.0	22.9	0.6–167.9	<0.05

Abbreviations: NS, not significant. Unk, unknown.

**Fig 4:**
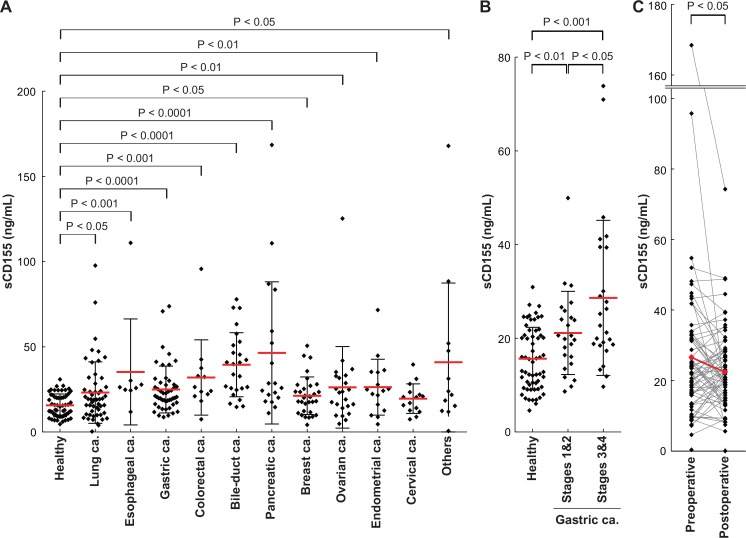
Soluble CD155 levels in sera of patients with various cancers. (**A**) Soluble CD155 levels in sera according to the various cancer types (lung: n = 52, esophageal: n = 8, gastric: n = 49, colorectal: n = 12, bile-duct: n = 25, pancreatic: n = 18, breast: n = 32, ovarian: n = 23, endometrial: n = 16, cervical: n = 15) analyzed by sandwich ELISA. Red bar: mean, black bar: SD. (**B**) Soluble CD155 levels in sera of gastric cancer patients stratified by disease stage. Stages 1&2: n = 24, stages 3&4: n = 25. (**C**) Soluble CD155 levels in sera of cancer patients who underwent surgery (n = 73) were analyzed for cancer before and after. Soluble CD155 level significantly decreased after operation. Red plot: mean, preoperative mean: 26.529 ng/mL, postoperative mean: 22.390 ng/mL, P < 0.05. Postoperative days: 69.30 ± 61.95.

Compared with those in healthy control samples, the sCD155 levels in sera from patients with each type of cancer except cervical cancer were significantly higher (lung: P < 0.05, esophageal: P < 0.001, gastric: P < 0.0001, colorectal: P < 0.001, bile-duct: P < 0.0001, pancreatic: P < 0.0001, breast: P < 0.05, ovarian: P < 0.01, endometrial: P < 0.01) (Table 1, Fig 4A). Notably, compared with healthy donors, patients with early stage (stages 1 and 2) gastric cancer had significantly higher sCD155 levels (P < 0.01). Furthermore, the sCD155 levels were significantly higher in patients with advanced stage (stages 3 and 4) gastric cancer than in patients in early stages (P < 0.05) and healthy controls (P < 0.001) (Fig 4B). Although sCD155 levels were not changed or even increased in certain patients after surgical resection of cancers and this might be dependent on each patient with different cancer, statistical analysis for total population demonstrated that sCD155 levels were significantly decreased (P < 0.05) (Fig 4C).

### sCD155 levels in sera were dependent on tumor burden in a mouse model

**Fig 5:**
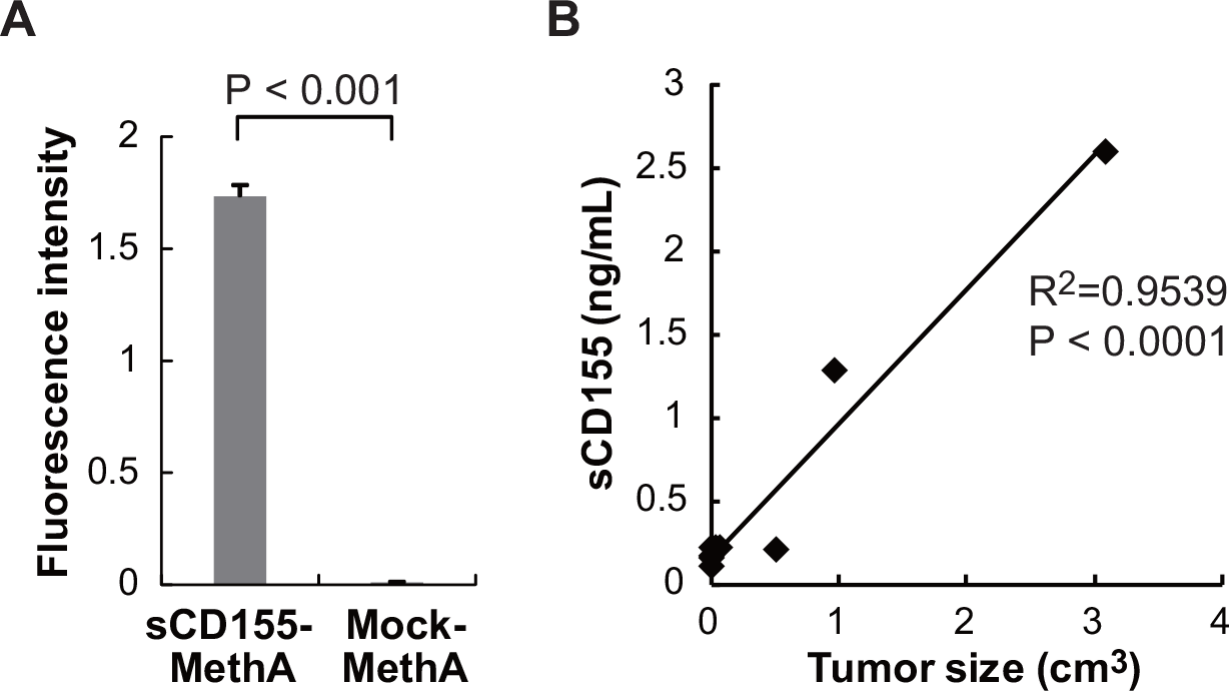
sCD155 levels in sera were dependent on tumor size in a mouse model. (**A**) MethA transfectant secreting sCD155 (sCD155-MethA) or control protein (Mock-MethA) were inoculated into peritoneal cavity and then ascites was collected. sCD155 levels in ascites of mice that had been inoculated with these transfectantss were measured by ELISA. (**B**) BALB/c mice were inoculated subcutaneously with sCD155-MethA transfectant. Tumor size and sCD155 levels in serum were measured and the correlation coefficient was calculated.

Based on the results described above, we hypothesized that sCD155 levels in the sera of cancer patients associate with tumor burden. To address this hypothesis, we transfected MethA fibrosarcoma cell line, which expressed only endogeneous mCD155, with a retrovirus vector containing either cDNA encoding the extracellular portion of mouse CD155 or with a mock control vector. We inoculated the transfectants (sCD155-MethA or Mock-MethA) into the peritoneal cavity of mice and, 10 days later, collected ascites. To quantify sCD155 level, we established an ELISA system. Although sCD155 was hardly detected in the ascites of mice that had been inoculated with Mock-MethA by ELISA, we detected a significant amount of sCD155 in the ascites of mice that had been inoculated with sCD155-MethA (Fig 5A). These results demonstrated that sCD155-MethA produced sCD155 *in vivo*. We then subcutaneously inoculated mice with sCD155-MethA and measured both serum sCD155 levels and tumor size. We observed that sCD155 levels in the sera were correlated with sCD155-MethA tumor size (Fig 5B). These results suggest that serum sCD155 levels associate with tumor burden.

## Discussion

Although sCD155 were identified 25 years ago [20], little is known about its expression and function. Here, we showed that the expression of sCD155 (*CD155β*) as well as mCD155 (CD155*a*) mRNA in tumor tissues was significantly higher than in non-tumor tissues. In addition, patients with various types of cancers showed significantly higher levels of serum sCD155, compared with healthy control samples. Moreover, sCD155 levels in sera were correlated with the disease progression in gastric cancer patients and tumor size in mice. Our results suggest that sCD155 levels in the sera of cancer patients are possibly dependent on tumor burden. Previous report showed that up-regulation of mouse CD155 is mediated by the Raf-MEK-ERK-AP-1 signaling pathway [23], suggesting that expression of both mCD155 and sCD155 is up-regulated through this pathway also in human, although further studies are required to determine how sCD155 is upregulated in cancers. Nonetheless, our results suggest that serum sCD155 level is potentially useful as a biomarker for cancer progression.

Although mCD155 expressed on tumors has been thought to be involved in DNAM-1-mediated tumor immunity, contradictory results have been reported. For example, overexpression of mCD155, as determined by immunohistochemical study by using anti-CD155 antibody, in lung adenocarcinoma and melanoma was correlated with poor prognosis [24, 25]. However, these studies did not discriminate between mCD155 and sCD155. In this study, we showed that the expression of both mCD155 (*CD155α*) and sCD155 (*CD155β*) were upregulated in cancers, including colorectal, gastric and breast cancers, compared with non-cancer tissues. Although, due to limited number of each type of cancer, we could not evaluate the correlation between expression levels of sCD155 (*CD155β*) mRNA in cancer tissues or serum CD155 levels and disease progression and prognosis, higher levels of sCD155 in the sera was associated with advanced stage of gastric cancer and tumor size in mice.

Previous reports demonstrated the role of soluble form of membrane receptors in tumor evasion. Membrane-bound molecules such as NKG2D ligands MHC class I-related molecule (MIC) and UL16-binding proteins (ULBPs) and Fas, which are involved in NK cells and CTL-mediated cytotoxicity against tumors, have been shown to be released as soluble forms in the sera of various cancer patients [26–30]. Unlike CD155, soluble MIC and ULBPs are generated from tumors by proteolytic shedding [26, 31, 32]. Tumors can evade from NKG2D-mediated immune surveillance by several mechanisms; one of them is that soluble MIC downregulates NKG2D expression [31, 33]. Previous report showed that high levels of soluble ULBP2 in sera were associated with poor disease prognosis in melanoma patients [27], suggesting that soluble ULBP2 is involved in tumor immune evasion.

In contrast to NKG2D ligands, the functional significance of sCD155 in tumor immunity has remained unclear. Recent studies revealed that DNAM-1 shares the ligand CD155 with T cell immunoreceptor with Ig and ITIM domains (TIGIT) or CD96 [34, 35]. Cross-linking CD96 with plate-bound CD155-Fc fusion protein inhibited the production of IFN*γ* by NK cells [36]. Moreover, CD96-deficient mice showed decreased experimental tumor metastasis [36], suggesting that CD96 and DNAM-1 oppose each other in tumor immunity. Similarly, TIGIT has an opposite effect to DNAM-1 on tumor immunity [37, 38]. To clarify the functional significance of sCD155 in tumor immunity, further studies are required how the interaction of sCD155 with its activating (DNAM-1) and inhibitory (CD96 and TIGIT) receptors and their signaling via these activating and inhibitory receptors are regulated.

## Supporting Information

S1 Fig Establishment of sandwich ELISA for sCD155. (**A**) Standard curve of human CD155-Fc fusion protein. (**B**) In a 12-well plate, 1 × 10^6^ HeLa were cultured per 1 mL of medium; culture supernatants were collected after 24 and 48 h for soluble CD155 protein detection.
(EPS)
